# Increased serum myeloid-related protein 8/14 level is associated with atherosclerosis in type 2 diabetic patients

**DOI:** 10.1186/1475-2840-10-41

**Published:** 2011-05-18

**Authors:** Wen Hui Peng, Wei Xia Jian, Hai Ling Li, Lei Hou, Yi Dong Wei, Wei Ming Li, Ya Wei Xu

**Affiliations:** 1 Department of Cardiology, Shanghai Tenth People's Hospital, Tongji University School of Medicine, Shanghai 200072, China; 2 Department of Endocrinology, Xinhua hospital, Shanghai Jiaotong University School of Medicine, Shanghai 200025, China

**Keywords:** MRP8/14, Diabetes mellitus, Coronary artery disease, Intima media thickness

## Abstract

**Background:**

Myeloid-related protein 8/14 (MRP8/14) is a stable heterodimer formed by two different calcium-binding proteins (MRP8 and MRP14). Studies have identified that MRP8/14 regulates vascular inflammation and serves as a novel marker of acute coronary syndrome. In this study, we evaluated the correlation between serum levels of MRP8/14, hsCRP, endogenous secretory receptor for advanced glycation end-products (esRAGE) and the occurrence of coronary artery disease (CAD), or carotid intima-media thickness (IMT) when CAD was not yet developed in diabetic patients.

**Methods:**

Serum levels of MRP8/14, esRAGE and hsCRP were measured in 375 diabetic patients. Then the results of those who had CAD were compared against who had not. Also, we investigated the associations between above-mentioned indicators and IMT of subjects without CAD in both diabetic group and non-diabetic one.

**Results:**

Serum MRP8/14 was significantly higher in CAD than in non-CAD group (9.7 ± 3.6 ug/ml vs. 8.2 ± 3.0 ug/ml, *P *< 0.001). It was associated with severity of CAD (*r *= 0.16, *P *= 0.026). In non-CAD group, MRP8/14 was associated with IMT in patients with (*r *= 0.30, *P *< 0.001) or without diabetes (*r *= 0.26, *P *= 0.015). The areas under the curves of receiver operating characteristic for CAD were 0.63 (95% CI 0.57-0.68) for MRP8/14, 0.76 (95% CI 0.71-0.81) for hsCRP and 0.62 (95% CI 0.56 -0.67) for esRAGE.

**Conclusion:**

In summary, we report that diabetic patients with CAD had elevated plasma MRP8/14 levels which were also positively correlated with the severity of CAD and carotid IMT in patients without clinically overt CAD.

## Introduction

Diabetes mellitus (DM) has been regarded as an important risk factor of cardiovascular diseases, and cardiovascular disease accounts for almost 70% deaths of diabetic patients [1]. The increased oxidative stress and inflammation are believed to be the pivotal mechanisms for diabetic cardiovascular complications [2].

Myeloid-related protein 8/14 (MRP8/14) is a stable heterodimer formed by two calcium-binding proteins (MRP8 and MRP14, also termed as Calgranulin A and Calgranulin B or S-100 calcium binding protein A8 and S100 calcium binding protein A9, respectively) [3]. MRP8 and MRP14, belonging to the S100 protein family, are expressed in activated human granulocytes and macrophages in inflammatory lesions, and have multiple functions such as activating NADPH oxidase [4-6], toll-like receptor 4 and receptor for advanced glycation endproducts (RAGE) [7,8] which are vital signaling pathways involved in the pathogenesis of micro- and macro-vascular complications in diabetes.

As mentioned above, MRP8/14 is involved in diabetic vascular complications, thus it is reasonable to propose that serum level of MRP8/14 would be a valuable index in assessing the risk of diabetic patients with developed cardiovascular diseases. Therefore, we investigated the association between the serum level of MRP8/14 and incidence of angiographically confirmed coronary artery disease (CAD) in diabetic patients. Meanwhile, we also attempted to explore the relation of serum MRP8/14 and carotid intima-media thickness (IMT) in people without CAD regardless of the presence of DM, with IMT being an early diagnostic index of atherosclerosis [9].

Being identified as a ligand for RAGE [10], MRP8/14 is believed to facilitate the detrimental effects of RAGE through advanced end glycation products [11]. Endogenous secretory RAGE (esRAGE) is the C truncated isoform of RAGE, which is able to prevent advanced glycation endproducts injury by acting as a decoy [12]. Previous studies of both our group and other researchers had shown that serum esRAGE level decreased in patients with diabetes and cardiovascular disease [13-15]. Therefore we also evaluated the serum levels of esRAGE and high sensitivity C reactive protein (hsCRP) in diabetic patients so as to determine their roles in inflammation and vascular damage.

## Methods

All subjects were local residents of Han ethnicity in Shanghai. The protocol was approved by the hospital's Ethics Committee and written informed consents were obtained from all participants. This study included 375 consecutive patients with T2DM and 87 patients without T2DM or CAD in Department of Cardiology of Shanghai Tenth People's Hospital (faciliated to Tongji University, School of Medicine) and Department of Endocrinology of Xinhua Hospital (faciliated to Shanghai Jiaotong University, School of Medicine). Patients without angiographically significant coronary stenosis were recruited for carotid ultrasound examination. Type 2 diabetes mellitus was diagnosed according to WHO standard or current intake of hypoglycemic agents [16]. Hypertension was defined as blood pressure ≥140/90 mmHg or having a history of receiving antihypertensive therapy. Those who had been diagnosed with acute myocardial infarction, systemic inflammatory disorders, severe heart failure (left ventricular ejection fraction <30%), advanced renal insufficiency or malignant tumor were excluded.

### Coronary angiography and quantitative analysis

Coronary angiography was performed by using standard Judkins technique or through a radial approach. Significant CAD was defined as the presence of luminal diameter stenosis ≥50% in the left anterior descending artery, left circumflex artery, right coronary artery, or their main branches. Left main trunk stenosis (≥50% luminal narrowing) was considered as a 2-vessel disease.

For coronary lesion analysis, we used quantitative coronary analyses (QCA, Centricity Cardiology CA 1000. v1.0, USA) as mentioned before [17]. To put it briefly, end-diastolic frames from angiograms were selected with identical angulations that best showed the stenosis at its most severe degree with minimal foreshortening and branch overlap. Arterial segments were defined from the images acquired on the basis of the anatomic location of proximal and distal side branches. The coronary artery segments analysis included all those plaques with a reference diameter ≥1.5 mm and a stenosis ≥20%. Using the outer diameter of the contrast-filled catheter as the calibration, the minimal lumen diameter in diastole was measured. Coronary artery score was calculated from per-patient average of the minimal lumen diameter of all the measured segments in observed coronary artery, and cumulative coronary obstruction was the sum of all percent diameter stenoses in standard index unit (50% = 0.50) [18].

### Carotid artery ultrasound

The carotid ultrasound examination was carried out by using Vivid-7 system (GE Vingmed Sound AS, Horton, Norway) with a scanning frequency of 5-12 MHz in B-mode. Operator was blind to the study group and did the ultrasound exam following the same protocol. The flow divider between the internal and external carotid arteries was identified, and the common carotid arteries were explored starting from 1 cm below the flow divider. Measurements were obtained by tracing the leading edge of the lumen-intima and the media-adventitia interfaces. Maximum right and left IMTs were averaged to obtain the carotid IMT measurement.

### Biochemical investigations

Blood samples were collected after overnight fasting in hospital and stored at -80°C. Serum glucose and lipid profiles (total cholesterol, low-density lipoprotein-cholesterol, high-density lipoprotein cholesterol, lipoprotein (a) and triglycerides) were measured (HITACHI 912 Analyser, Roche Diagnostics, Germany). Serum concentrations of glycosylated hemoglobin A1c (HbA1c), blood urea nitrogen (BUN), creatinine, and uric acid were assessed by standard methods. Renal function was evaluated according to the estimated creatinine clearance rate (CrCl) derived from Cockroft-Gault formula, where CrCl (ml/min) = ([140-age] × weight [kg])/(serum creatinine [mg/dl] × 72), corrected in women by a factor of 0.85 [19]. HsCRP and esRAGE were determined by ELISA kit as indicated earlier [15]. MRP8/14 level was also assessed using ELISA kit (Bühlmann Laboratories, Schönenbuch, Switzerland).

### Statistical analysis

Continuous variables were expressed as mean ± standard deviation (SD), and categorical variables were presented as frequencies. Normal distribution was evaluated by Kolmolgorov-Smirnov test. Logarithmic transformation was performed on the continuous variables of non-normal distribution before statistical calculations that requested normal distribution. Comparisons between groups were made using unpaired *t *tests, analysis of variance or nonparametric Mann-Whitney U test, when appropriate. Qualitative variables were compared using Chi-square test. Pearson's correlations were used to test the relationship between continuous variables. Receiver operating characteristic (ROC) analysis was also carried out on the serum levels of the MRP8/14, hsCRP and esRAGE for the differentiation of CAD. Multivariate logistic regression model was constructed to assess the independent determinants of CAD. A 2-sided probability level of ≤0.05 was considered significant. All analyses were done with SPSS for Windows 13.0 (SPSS Inc, Chicago, Illinois).

## Results

### Baseline characteristics

General characteristics of patients were shown in Table 1. Patients in CAD and non-CAD groups had similar waist-to-hip ratio, systolic blood pressure, diastolic blood pressure, diabetic duration and proportion of smoking, hypertension, and stroke. But CAD patients were older and had a higher percentage of male than non-CAD group. As for lipid profile, CAD patients had higher serum lipoprotein (a) and lower HDL levels (*P *< 0.05). There were insignificant differences in fasting glucose and 2 h postprandial glucose between two groups. CAD group had worse renal function (33.5 ± 14.6 ml/min vs. 44.2 ± 18.6 ml/min, *P *< 0.001). Serum esRAGE in CAD group was lower, while hsCRP and MRP8/14 were significantly higher than non-CAD group (0.26 ± 0.18 ng/ml vs. 0.31 ± 0.16 ng/ml for esRAGE; 15.4 ± 15.9 mg/L vs. 6.7 ± 11.1 mg/L for hsCRP; 9.7 ± 3.6 ug/ml vs. 8.2 ± 3.0 ug/ml for MRP8/14, all *P *< 0.05).

**Table 1:** Clinical characteristics

	No CAD	CAD	*P*
Age (years)	64 ± 10	68 ± 10	<0.001
Sex (men, %)	90 (52.9%)	130 (63.4%)	0.04
BMI (kg/m2)	25.7 ± 4.0	25.0 ± 3.2	0.037
Waist-to-hip ratio	0.93 ± 0.07	0.92 ± 0.08	NS
Systolic BP (mmHg)	137 ± 19	139 ± 23	NS
Diastolic BP (mmHg)	79 ± 11	78 ± 12	NS
Smoker (n, %)	44 (25.9%)	51 (24.9%)	NS
Hypertension (n, %)	113 (66.5%)	155 (75.6%)	0.051
Stroke (n, %)	17 (10.0%)	20 (9.8%)	NS
Duration of diabetes (years)	8 ± 7	8 ± 7	NS
Total Cholesterol (mmol/L)	4.55 ± 1.08	4.41 ± 1.16	NS
Triglyceride (mmol/L)	1.98 ± 1.70	1.98 ± 1.45	NS
HDL Cholesterol (mmol/L)	1.19 ± 0.32	1.14 ± 0.34	0.030
LDL Cholesterol (mmol/L)	2.73 ± 0.78	2.64 ± 0.91	NS
lipoprotein (a) (mg/L)	102 ± 158	238 ± 186	<0.001
BUN (mmol/L)	6.3 ± 2.6	7.2 ± 6.2	NS
Creatinine (umol/L)	74.5 ± 28.1	91.1 ± 39.6	<0.001
CrCl (ml/min)	44.2 ± 18.6	33.5 ± 14.6	<0.001
Uric acid (mg/L)	74.5 ± 28.1	91.1 ± 39.6	0.034
Fasting glucose (mmol/L)	7.7 ± 2.5	7.3 ± 2.7	NS
2 h Postprandial glucose (mmol/L)	13.1 ± 4.7	12.9 ± 4.2	NS
HbA1c (%)	8.1 ± 2.0	7.6 ± 1.4	0.012
Fasting insulin (mU/L)	10.1 ± 10.8	8.3 ± 7.8	NS
2 h Postprandial insulin (mU/L)	48.2 ± 48.7	39.4 ± 35.5	NS
hsCRP (mg/L)	6.7 ± 11.1	15.4 ± 15.9	<0.001
esRAGE (ng/ml)	0.31 ± 0.16	0.26 ± 0.18	<0.001
MRP8/14 (ug/ml)	8.2 ± 3.0	9.7 ± 3.6	<0.001

Data are presented as mean ± SD or number (percent).

BMI, body mass index; HDL, high-density lipoprotein cholesterol; LDL, low-density lipoprotein cholesterol; BUN, blood urea nitrogen; CrCl: creatinine clearance; HbA1c, hemoglobin A1c; hsCRP, high sensitive C-reactive protein; esRAGE, endogenous secretory receptor for advanced glycation end products; MRP8/14, Myeloid-related protein 8/14

### The relationship between MRP8/14 and diabetic CAD

We compared the diagnostic value for CAD of serum MRP8/14 complex level with esRAGE and hsCRP levels in T2DM patients by ROC curves. The areas under the curves were 0.63 (95% confidence interval (CI): 0.57-0.68) for MRP8/14, 0.77 (95% CI: 0.72-0.81) for hsCRP and 0.62 for esRAGE (95% CI: 0.56-0.67). Given a cut-off value of 10.9 mg/ml for MRP8/14, serum MRP8/14 complex level was able to significantly distinguish CAD patients among diabetic subjects, with sensitivity of 38.5% and specificity of 85.3% (*P *< 0.05).

In multivariate logistic regression analysis, serum MRP8/14 was significantly associated with the presence of CAD (OR = 1.13, 95% CI [1.050-1.225], *P *= 0.001), and it was independent of traditional risk factors for CAD (see Table 2).

**Table 2:** Multivariable regression analysis of independent determinants of CAD in Type 2 diabetes mellitus

	Odds ratio	95% Confidence interval	*P*
Age	1.02	0.99-1.05	NS
Male	1.49	0.84-2.65	NS
Body mass index	0.926	0.863-0.994	0.034
Smoking	0.983	0.531-1.820	NS
Hypertension	1.624	0.925-2.851	NS
Triglyceride	1.035	0.877-1.222	NS
Total Cholesterol	0.966	0.703-1.327	NS
LDL Cholesterol	0.960	0.651-1.415	NS
HDL Cholesterol	0.696	0.319-1.517	NS
Creatinine	1.009	1.00-1.02	NS
hsCRP	1.10	1.061-1.138	<0.001
esRAGE	0.211	0.054-0.826	0.025
MRP8/14	1.13	1.050-1.225	0.001

HDL, high-density lipoprotein cholesterol; LDL, low-density lipoprotein cholesterol; hsCRP: high-sensitivity C-reactive protein; esRAGE, endogenous secretory receptor for advanced glycation end products; MRP8/14, Myeloid-related protein 8/14

### Relationship of MRP8/14 with clinical, biochemical parameters, and CAD severity

Correlation analyses were performed to evaluate potential associations between serum MRP8/14 level and clinical measurements. A significant linear correlation existed between serum MRP8/14 level and waist-to-hip ratio, renal function, hsCRP level, respectively, in the population studied (see Table 3, all *P *< 0.05).

**Table 3:** Relation of MRP8/14 with other parameters

	*r*	*P*
Body mass index	0.05	NS
Waist-to-hip ratio	0.11	0.030
Total Cholesterol	-0.03	NS
Triglyceride	0.03	NS
HDL Cholesterol	-0.01	NS
LDL Cholesterol	-0.02	NS
lipoprotein (a)	0.10	0.055
BUN	0.11	0.030
Creatinine	0.14	0.008
Uric acid	-0.02	NS
Fasting glucose	-0.01	NS
2 h Postprandial glucose	0.00	NS
HbA1c (%)	0.01	NS
Fasting insulin	-0.05	NS
2 h Postprandial insulin	-0.04	NS
hsCRP	0.15	0.003
esRAGE	-0.05	NS
coronary artery score	0.23	0.009
cumulative coronary obstruction	0.21	0.010

HDL, high-density lipoprotein cholesterol; LDL, low-density lipoprotein cholesterol; BUN, blood urea nitrogen; HbA1c, hemoglobin A1c; hsCRP, high sensitive C-reactive protein; esRAGE, endogenous secretory receptor for advanced glycation end products; MRP8/14, Myeloid-related protein 8/14

In CAD cases, serum MRP8/14 and hsCRP levels were positively associated with severity of CAD (*r *= 0.16, *P *= 0.026; *r *= 0.15, *P *= 0.028, respectively). However, esRAGE showed negative correlation with severity of CAD with weak significance (*r *= -0.12, *P *= 0.095) (See Table 4). In addition, we found MRP8/14 and hsCRP levels were also strongly associated with coronary artery score and cumulative coronary obstruction (*r *= 0.23, *P *= 0.009; *r *= 0.21, *P *= 0.010, respectively).

**Table 4:** Association of CAD severity with biochemistry characteristics

	1 vessel disease	2 vessel disease	3 vessel disease	4 vessel disease	*r *value	*P *value for trend
esRAGE (ng/ml)	0.29 ± 0.18	0.24 ± 0.18	0.26 ± 0.17	0.16 ± 0.06	-0.12	0.095
hsCRP (mg/L)	12.2 ± 10.2	14.8 ± 13.6	16.7 ± 17.2	22.3 ± 16.3	0.15	0.028
MRP8/14 (ug/ml)	9.0 ± 3.1	9.9 ± 3.9	10.3 ± 3.6	10.8 ± 4.0	0.16	0.026

Data are presented as mean ± SD

hsCRP: high-sensitivity C-reactive protein; esRAGE, endogenous secretory receptor for advanced glycation end products; MRP8/14, Myeloid-related protein 8/14

### Relationship of carotid IMT with MRP8/14 and other parameters

**Figure 1:**
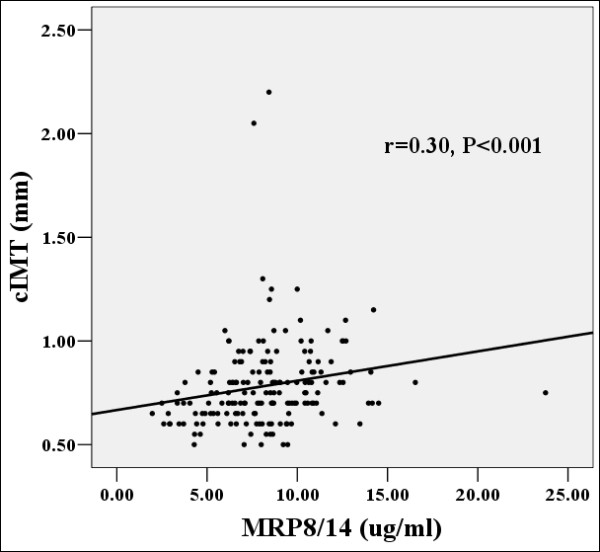
**Correlation between MRP8/14 concentration and carotid IMT in diabetic patients without CAD**. Positive correlation (Spearman's rho) was observed between serum MRP8/14 concentration and carotid intima-media thickness in diabetic patients without CAD, r = 0.30, P < 0.001. cIMT:carotid intima-media thickness; MRP8/14, Myeloid-related protein 8/14

In univariate analysis, MRP8/14 (*r *= 0.30, *P *< 0.001)(Figure 1), hsCRP (*r *= 0.23, *P *= 0.003), and 2 h postprandial glucose (*r *= 0.16, *P *= 0.032) levels were positively correlated with carotid IMT in non-CAD group. In multiple regression analysis after adjustment of age, sex, hypertension, etc., MRP8/14 and hsCRP, but not 2 h postprandial glucose level, were considered independent determinants for IMT in diabetic patients without CAD (*P *< 0.05).

In subjects with neither DM nor angiographically confirmed CAD, we also found positive correlation between carotid IMT and serum MRP8/14 level (*r *= 0.26, *P *= 0.015).

## Discussion

To date, there have been few reports focusing on the clinical importance of MRP8/14 in diabetic complications. In this study, we found that diabetic patients with angiographically confirmed CAD had elevated serum MRP8/14 level. Moreover, MRP8/14 was highly associated with not only the severity of CAD, but also the early atherosclerotic marker, the carotid IMT in patients without CAD.

### MRP8/14 and inflammatory vascular disease

Elevated serum MRP8/14 level was found in several chronic inflammatory conditions, including rheumatoid arthritis, allograft rejections, and inflammatory bowel and lung diseases [20-25]. MRP8/14 was not only involved in promoting the inflammatory response in infected lesions but also identified as a potent amplifier of inflammation during autoimmune reaction [10]. MRP8/14 was also reported to be expressed in human atherosclerotic plaques, especially in rupture-prone lesions [26]. Recent studies identified that MRP8/14 broadly regulated vascular inflammation and contributed to the biological response to vascular injury [27]. Clinical studies showed MRP8/14 as a novel marker of acute coronary syndromes and a predicting factor for future cardiovascular events [22,23,26].

Some studies suggested that MRP8/14 participated in diabetic vascular disease. Serum level of MRP8/14 was increased in type 1 diabetes mellitus thus enhanced adhesion of circulating monocytes to fibronectinin [28], and serum MRP8/14 level was found to be an indicator of microcirculatory defects in diabetic nephropathy [29]. In this study, we further confirmed that compared with non-CAD diabetic patients, serum MRP8/14 level was increased in patients with CAD. More interestingly, serum MRP8/14 was not only associated with severity of CAD, but also with carotid IMT in subjects without CAD. Measurement of carotid IMT was determined with good and comparable reproducibility in both subjects with T2DM and those without [30]. Patients with diabetes often develop CAD in their early stages, however the early diagnosis of CAD in diabetic patients is often missed as these patients are always asymptomatic or having atypical chest pain. The availability of a sensitive and specific early biomarker for diabetic atherosclerosis would be highly desirable for the diagnosis and therapeutic strategy of diabetes complications. The strong association between serum MRP8/14 and carotid IMT in patients without CAD further confirmed serum MRP8/14 as a novel marker for diabetic vasculopathy even in its early stage.

It should be noted that although MRP8/14 was strongly associated with diabetic vasculopathy, the diagnostic value of MRP8/14 for CAD was limited. In ROC analysis, the area under the curve of MRP8/14 was only 0.63, which was not superior to that of hsCRP. It may partly due to the medication of diabetic patients, such as statin and hypoglycemic drugs. Since our study was a cross-section study, the strong association between MRP8/14 and IMT and its clinical implication is yet to be confirmed by further cohorts study.

### The association of MRP8/14 with renal function and other serum factors

Another question which should be taken into account was that whether or not the elevated serum MRP8/14 level was due to worse renal function in CAD group. Previous study showed that serum MRP8/14 was related to diabetic nephropathy [29]. In our study, we also found strong association between MRP8/14 and CrCl. By multivariate regression analysis, we found that serum MRP8/14 was a determinant for CAD even after adjustment of renal function, which suggested that detrimental effect of MRP8/14 in macrovascular disease was independent of renal function.

Although serum MRP8/14 level was increased in diabetic microvascular or macrovascular diseases [29], previous studies did not find its increase in type 2 diabetes compared to that in normal subjects. In our study, we found no correlation between serum MRP8/14 and parameters related to glucose homeostasis both in univariate and multivariate regression analysis; nevertheless, serum MRP8/14 was positively associated with hsCRP. This suggested that the glucose homeostatic effects on serum MRP8/14 concentration were minimal and systemic inflammation should be the principal factor for regulating serum MRP8/14 expression [28]. Moreover, we did not find any association between serum MRP8/14 and HbA1c--the representative marker of systemic glycation, or serum esRAGE--a selective splice isoform of RAGE showing functions such as inhibiting atherogenesis and mitigating progression of atherosclerotic lesion in diabetic mice [31,32]. The lacking of association between MRP8/14 and glucose or glycation products further confirmed that regulation of serum MRP8/14 level in diabetic complications was independent of glucose metabolism.

## Conclusions

In summary, we report that diabetic patients with CAD had elevated plasma MRP8/14 levels which were also positively correlated with the severity of CAD and carotid IMT in patients without clinically overt CAD. Therefore, routine assessment of MRP8/14 could be recommended in diabetic patients who were considered as the high risk population of CAD, and this may provide physicians with another effective method of evaluating vascular risks in diabetic patients.

## Abbreviations

BUN: blood urea nitrogen; CAD: coronary artery disease; CI: confidence interval; CrCl: creatinine clearance; DM: diabetes mellitus; esRAGE: endogenous secretory receptor for advanced glycation endproducts; HbA1c: glycosylated hemoglobin A1c; hsCRP: high sensitivity C reactive protein; IMT: intima-media thickness; MRP8/14: myeloid-related protein 8/14; QCA: quantitative coronary analyses; SD: standard deviation;

## Competing interests

The authors declare that they have no competing interests.

## Authors' contributions

XYW designed, coordinated and wrote the manuscript. PWH coordinated and wrote the manuscript. PWH, JWX, and LHL carried out the sample collection. JWX performed ELISA. HL performed statistical analysis. HL, WYD, and LWM performed coronary angiography. All authors read and approved the final manuscript.
